# Supplementary material for: Toward Tumor Fight and Tumor Microenvironment Remodeling: PBA Induces Cell Cycle Arrest and Reduces Tumor Hybrid Cells’ Pluripotency in Bladder Cancer
Source: Cancers (Basel). 2022 Jan 7;14(2):287. doi: 10.3390/cancers14020287 (PMC8773853; doi:10.3390/cancers14020287)
Supplement: Supplementary file 1 [file cancers-14-00287-s001.zip › cancers-1549942-supplementary.pdf]

cancers MDPI

Supplementary Materials: Toward Tumor Fight and Tumor Microenvironment Remodeling: PBA Induces Cell Cycle Arrest and Reduces Tumor Hybrid Cells' Pluripotency in Bladder Cancer

Carolina Rubio, José Avendaño-Ortiz, Raquel Ruiz-Palomares, Viktoriya Karaivanova, Omaira Alberquilla, Rebeca Sánchez-Domínguez, José Carlos Casalvilla-Dueñas, Karla Montalbán-Hernández, Iris Lodewijk, Marta Rodríguez-Izquierdo, Ester Munera-Maravilla, Sandra P. Nunes, Cristian Suárez-Cabrera, Miriam Pérez-Crespo, Víctor G. Martínez, Lucía Morales, Mercedes Pérez-Escavy, Miguel Alonso-Sánchez, Roberto Lozano-Rodríguez, Francisco J. Cueto, Luis A. Aguirre, Félix Guerrero-Ramos, Jesús M. Paramio, Eduardo López-Collazo and Marta Dueñas

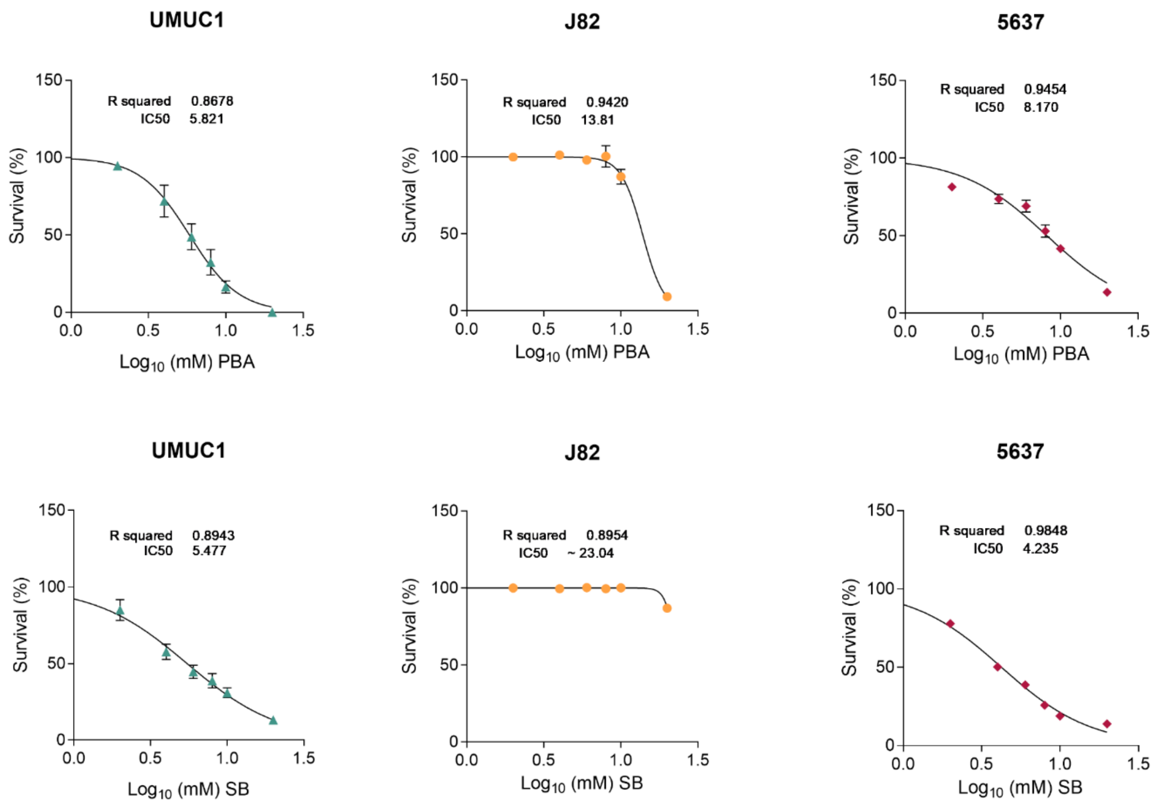

Figure S1. Summary of sensitivity assays of UMUC1, 5637 and J82 BC cells to PBA and SB. Data come from 5 independent experiments for each cell line and are shown as mean ± SEM for different concentrations of the compounds.

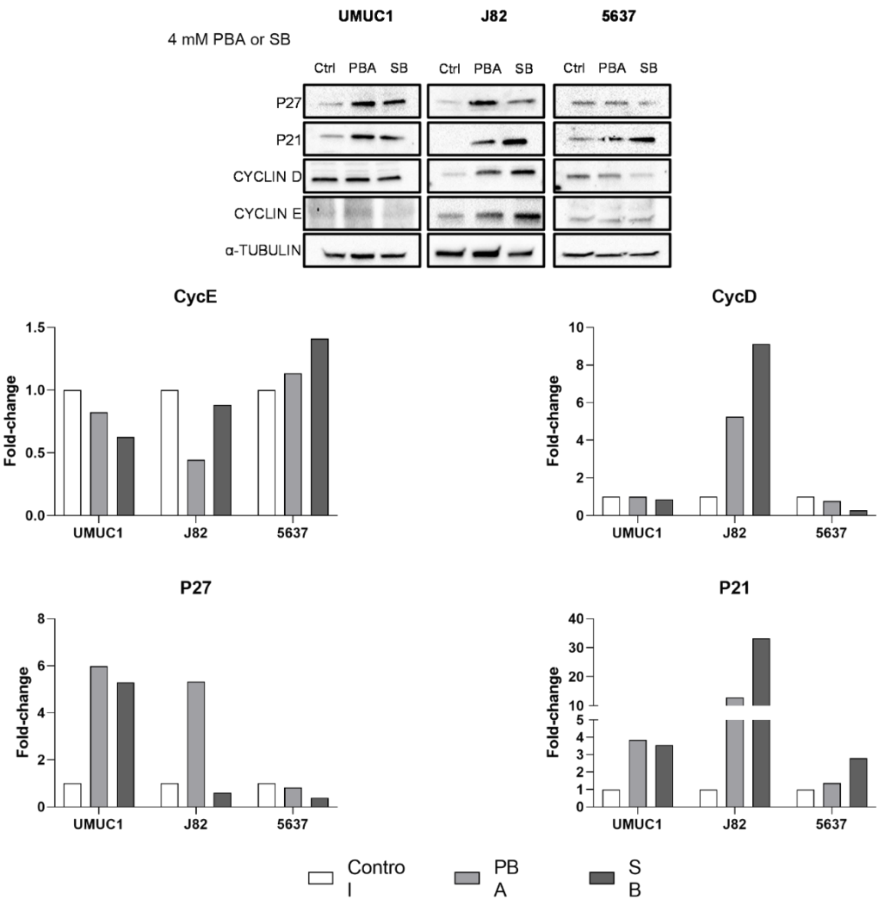

Figure S2. Expression of cell cycle related proteins in UMUCI, J82 and 5637 BC cells without treatment (Ctrl) and after treatment with PBA and SB. Quantification levels of all marks in the same cells is shown, α-TUBULIN was used for loading normalization.

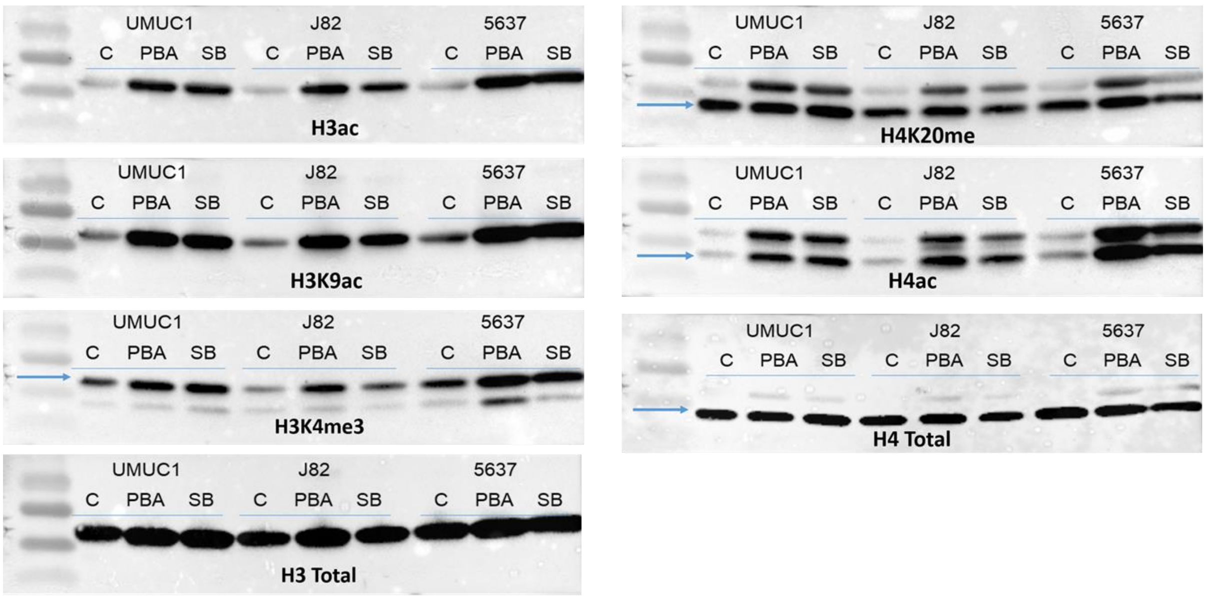

Figure S3. Original Western blots images.

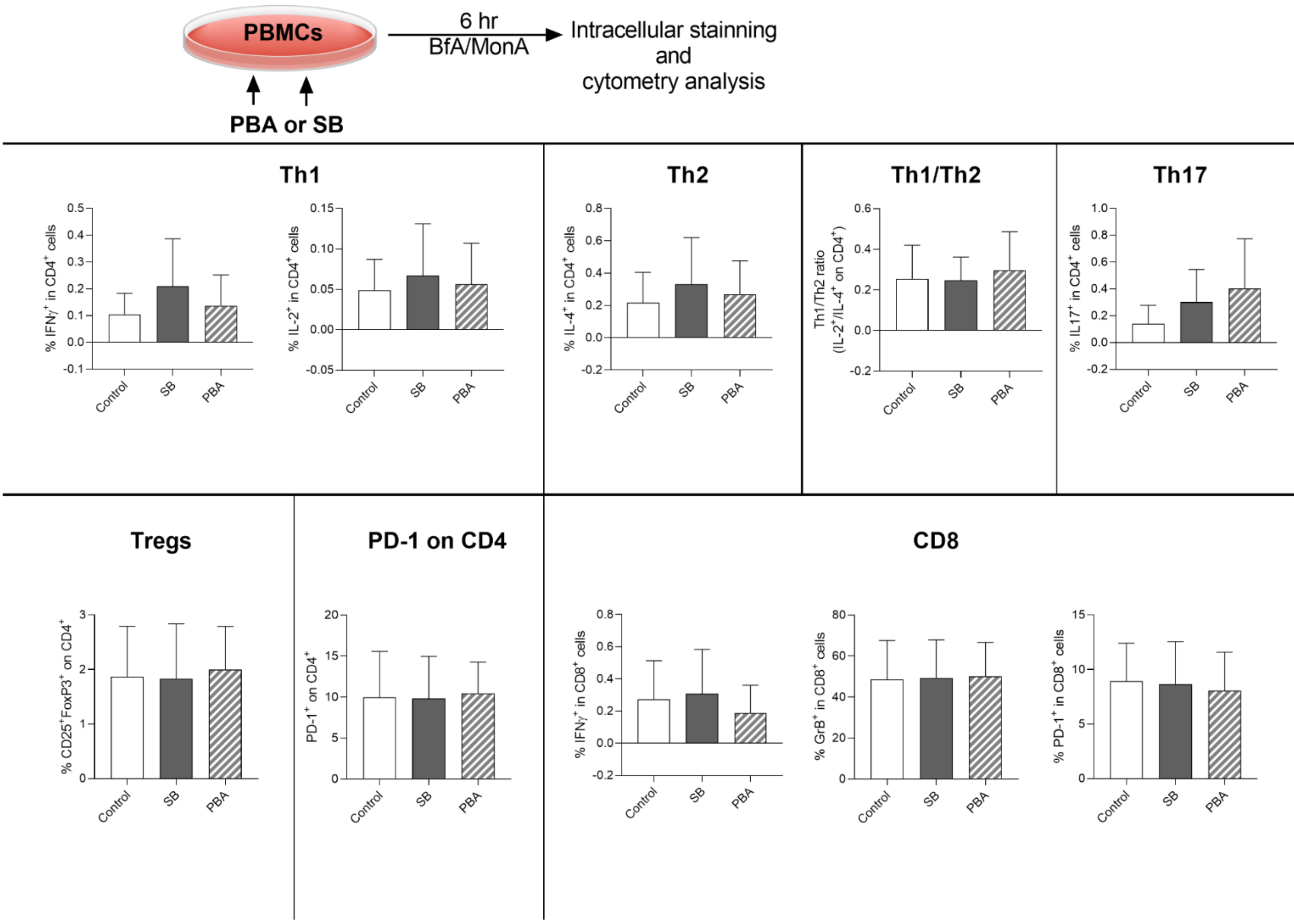

Figure S4. Citokyne expression profiles of PBMC treated with PBA and SB. Brefeldin A and Monensin were used to enhance cytokine detection.

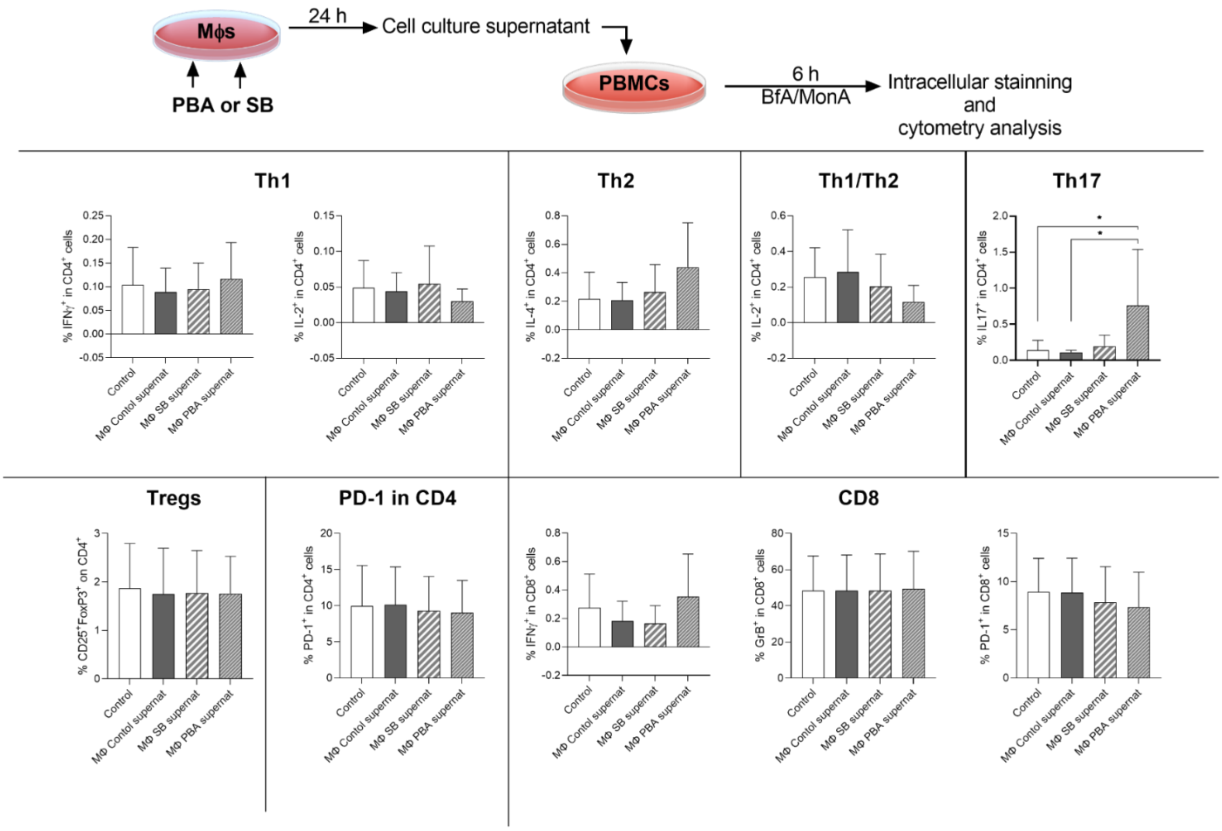

Figure S5. Citokyne expression profiles of PBMC stimulation with conditioned medium from macrophage treated with PBA and SB. Brefeldin A and Monensin were used to enhance cytokine detection.

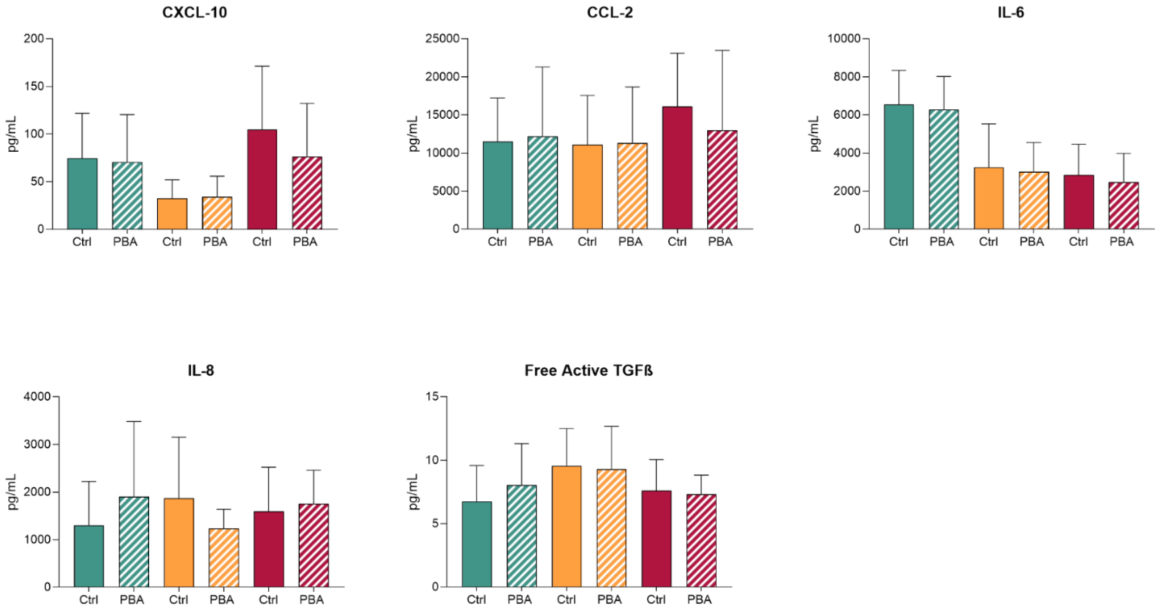

Figure S6. Evaluation of cytokine expression profiles characteristic for M1 or M2 macrophage polarization in co-culture supernatants. CXCL10, CCL2, IL-6, IL-8 and free active TGFb cytokine expression profiles are shown for single-culture and co-culture supernatants, Supernatants of macrophages and BC cell single cultures were included as a control.

Table S1. Antibodies employed in the present study.

Antibodies for Western Blot

Target	Clone	Source	Dilution
Acetyl-Histone H3 (Lys9)		Cell Signaling #9649	1:1000
H4K20me		Abcam9051	1:1000
H3K4me3		Abcam 8580	1:1000
Acetyl-H3	-	Millipore 06-599	1:1000
H3	96C10	Cell Signaling #3638	1:10000
Acetyl-H4	-	Millipore 06-598	1:100
H4	62-141-13	Millipore 05-858	1:1000
p27	-	Abcam ab7961	1:500
p21	F-5	Santa Cruz sc-6246	1:500
Cyclin D1	H-295	Santa cruz sc-753	1:200
Cyclin E	-	Upstate biotechnology 06-134	1:500
α-Tubulin	FL-335	Sigma T5168	1:1000

Secondary antibody for Western Blot

Target	Clone	Conjugated	Source	Dilution
Anti-Rabbit IgG	Donkey	HPR	GE Healthcare NA934	1:5000
Anti-Mouse IgG	Donkey	HPR	JACKSON Nº 715-035-151	1:5000

Antibodies for Flow Cytometry

Target	Clone	Source	Dilution
CD11b	-	Beckman Coulter IM0530	1 µl per million cells in 100 µl
EpCAM	9C4	BioLegend 324208	5 µl per million cells in 100 µl

List of antibodies for extracellular and intracellular staining for T cell stimulation by Flow Cytometry

Target	Clone	Source	Fluorochrome
Extracellular markers			
CD3	UCHT1	Biolegend 300436	BV570
CD4	SK3	Biolegend 344608	PerCP/Cy5.5
CD8	SK1	BD biosciences 612889	BUV805
CD25	2A3	BD biosciences 347643	FITC
PD-1	EH12.2H7	Biolegend 329929	BV785
Intracellular markers			
Granzyme B	QA16A02	Biolegend 372214	PE/Cy7
IFNγ	4S.B3	Biolegend 502540	BV711
FoxP3	236A/E7	BD biosciences 563955	PE-Dazzle594
IL-2	MQ1-17H12	BD biosciences 565136	APC-R700
IL-17	SCPL1362	BD biosciences 560439	Alexa Fluor 647
IL-4	MP4-25D2	BD biosciences 566274	BV421
TNFα	MAb11	BD biosciences 563418	BV650

Table S2. Primer sequences for RT-qPCR.

Primer name	Sequence 5' -> 3'
TBP-F	AGTGAAGAACAGTCCAGACTG
TBP-R	CCAGGAAATAACTCTGGCTCAT
TBP-RT	TGCCTTTGTTGCTCTT
CDK1NA(p21)-F	CCTCATCCCGTGTTCTCCTTT
CDK1NA(p21)-R	ACTTGTCCGCTGGGTGGTAC
CDK1NA(p21)-RT	GGTGAATTTCATAACCG
CDKN1B (p27)-F	CGCTTTGTTTTGTTCGGTTT
CDKN1B (p27)-R	CACTCGCACGTTTGACATCT
CDKN1B (p27)-RT	GTCCCGGGTTAACTCTTCGT
MYC-F	AATGAAAAGGCCCCCAAGGTAGTTATCC
MYC-R	GTCGTTTCCGCAACAAGTCCTCTTC
MYC-RT	GTTAGAAGGAATCG
E2F1-F	TCCAAGAACCACATCCAGTG
E2F1-R	CTGGGTCAACCCCTCAAG
E2F1-RT	GTATAAATTAAATGTTTCCA
BMP4-F	TCCACAGCACTG GTCTTG
BMP4-R	TGGGATGTTCTCCAGATG
BMP4-RT	GGG TG TGCTGAGGTTA
PD-L1-F	CCATACAGCTGAATTGGTCATC
PD-L1-R	CAGAATTACCAAGTGAGTCCTTTCA
PD-L1-RT	GTCTCCTCCAAA
TLR4-F	CCTGCGTGAGACCAGAAAG
TLR4-R	TTCAGCTCCATGCATTGATAA
TLR4-RT	TGTCAATATTAAGGTAGAGA
BMPR-2-F	TCTGGATCTTTCAGCCACAA
BMPR-2-R	TGCCATCTTGTGTTGACTCAC
BMPR-2-RT	AGTGGAGATGACCC
SOX2-F	CGGAGGAGAAGGAAAGTCG
SOX2-R	CCAGCAAACTCCAGCAGAC
SOX2-RT	GCT TGGAGACTAGC
NANOG-F	ACCTTCCAATGTGGAGCAAC
NANOG-R	ACTGGATGTTCTGGGTCTGG
NANOG-RT	GTTGGTCAGCACAGGAGAAAATGCC
IL-10-F	GATGCCTTCAGCAGAGTGAA
IL-10-R	GCAACCCAGGTAACCCTTAAA
IL-10-RT	GTCTGGGTCTT

GTCTGGGTCTT
